# How different attributes are weighted in professionals’ decision-making in Pediatric Dentistry—a protocol for guiding discrete choice experiment focused on shortening the evidence-based practice implementation for dental care

**DOI:** 10.1186/s12903-024-04090-3

**Published:** 2024-04-19

**Authors:** Gabriela Manco Machado, Ana Clara Falabello Luca, Renata Paz Leal Pereira, Ana Yne Fernandez, Lucas Gabriel Santini Rodrigues, Isabella Petroline Leite, Maximiliano Sergio Cenci, Tatiana Pereira Cenci, Ana Paula Pires Santos, Branca Heloisa Oliveira, Paulo Nadanovsky, Marina Deus Moura Lima, Marcoeli Silva Moura, Edson Hilan Gomes Lucena, Tathiane Larissa Lenzi, Ana Carla Crispim, Fernanda Campos Almeida Carrer, Mariana Gabriel, Claudia Cazal Lira, Carla Vecchione Gurgel, Helder Henrique Costa Pinheiro, Gilberto Alfredo Pucca, Fábio Carneiro Martins, Paola Gondim Calvasina, Maria Fernanda Montezuma Tricoli, Camila Menezes Costa Castelo Branco, Raiza Dias Freitas, José Carlos Pettorossi Imparato, Daniela Prócida Raggio, Tamara Kerber Tedesco, Fausto Medeiros Mendes, Mariana Minatel Braga

**Affiliations:** 1 Orthodontics and Pediatric Dentistry Department, Faculty of Dentistry, University of São Paulo, Av. Prof. Lineu Prestes, 2227 - Butantã, São Paulo, SP 05508-000 Brazil; 2 Instituto Ayrton Senna, São Paulo, Brazil; 3 Department of Dentistry, Radboud University Medical Center, Radboud Institute for Health Sciences, Nijmegen, The Netherlands; 4 Department of Community and Preventive Dentistry, University of the State of Rio de Janeiro, Rio de Janeiro, Brazil; 5 Department of Epidemiology, Institute of Social Medicine, Rio de Janeiro State University, Rio de Janeiro, Brazil; 6 Department of Epidemiology, National School of Public Health, Oswaldo Cruz Foundation, Rio de Janeiro, Brazil; 7 Department of Pathology and Dental Clinic, Federal University of Piauí, Teresina, Piauí Brazil; 8 Department of Clinical and Social Dentistry, Federal University of the Paraiba, Paraiba, Brazil; 9 Department of Surgery and Orthopedics, Faculty of Dentistry, Federal University of Rio Grande do Sul, Porto Alegre, Brazil; 10 Academic Area of Pathology, Federal University of Pernambuco, Recife, Brazil; 11 Faculty of Dentistry, Federal University of Bahia, Salvador, Brazil; 12 Faculty of Dentistry, Federal University of Pará, Belem, Brazil; 13 Faculty of Dentistry, University of Brasilia, Brasilia, Brazil; 14 Faculty of Dentistry, State University of Ceara, Fortaleza, Brazil; 15 Oral Health Technical Area, State Health Department, São Paulo, Brazil; 16 Oral Health Coordination, João Pessoa, Paraiba, Brazilth System, João Pessoa, Brazil; 17 Pontifícia Universidade Católica Madre y Maestra, Santo Domingo, Dominican Republic; 18 Universidade Galileo, Botucatu, SP, Brazil

**Keywords:** Preference, Discrete choice experiments, Dentistry

## Abstract

**Background:**

Important evidence has been constantly produced and needs to be converted into practice. Professional consumption of such evidence may be a barrier to its implementation. Then, effective implementation of evidence-based interventions in clinical practice leans on the understanding of how professionals value attributes when choosing between options for dental care, permitting to guide this implementation process by maximizing strengthens and minimizing barriers related to that.

**Methods:**

This is part of a broader project investigating the potential of incorporating scientific evidence into clinical practice and public policy recommendations and guidelines, identifying strengths and barriers in such an implementation process. The present research protocol comprises a Discrete Choice Experiment (DCE) from the Brazilian oral health professionals’ perspective, aiming to assess how different factors are associated with professional decision-making in dental care, including the role of scientific evidence. Different choice sets will be developed, either focusing on understanding the role of scientific evidence in the professional decision-making process or on understanding specific attributes associated with different interventions recently tested in randomized clinical trials and available as newly produced scientific evidence to be used in clinical practice.

**Discussion:**

Translating research into practice usually requires time and effort. Shortening this process may be useful for faster incorporation into clinical practice and beneficial to the population. Understanding the context and professionals’ decision-making preferences is crucial to designing more effective implementation and/or educational initiatives. Ultimately, we expect to design an efficient implementation strategy that overcomes threats and potential opportunities identified during the DCEs, creating a customized structure for dental professionals.

**Trial registration:**

https://osf.io/bhncv.

**Supplementary Information:**

The online version contains supplementary material available at 10.1186/s12903-024-04090-3.

## Background

There has been a continuous production of important evidence that needs to be translated into practice. However, there is a gap between what is produced and what is practised [1, 2]. Translation of research into products, policies, and practices is estimated to take 17 years [2]. Traditional decision-making is entrusted to health professionals. It is a complex process that can be affected by many factors, such as those related to the patients (as socioeconomic status, patient age and gender), to the environment (resources available and geographic location), and to the professional itself (professional age, gender, work overload, family issues, beliefs, school philosophy) [3–9].

Although evidence-based practice has been widely recommended [10], the actual role of scientific evidence in decision-making and its relevance (or remains) when combined with other decisive factors is still unclear. Indeed, we do not properly know which attributes (e.g. costs, training, success rates, patients’ satisfaction) are considered by oral health professionals when choosing between options for caring for their patients. Professionals seem to use their own criteria to identify and compare/weigh the options [11]. Therefore, professional consumption of scientific evidence, especially recently produced, may hinder its implementation, increasing the time lapse between evidence production and its use in the real world. Then, implementation research should plan actions to change the beliefs and paradigms of these professionals [12] or customize their actions to offer the evidence in the “right” format for its potential consumers, improving the engagement of professionals with scientific evidence through knowledge translation initiatives.

**Fig. 1:**
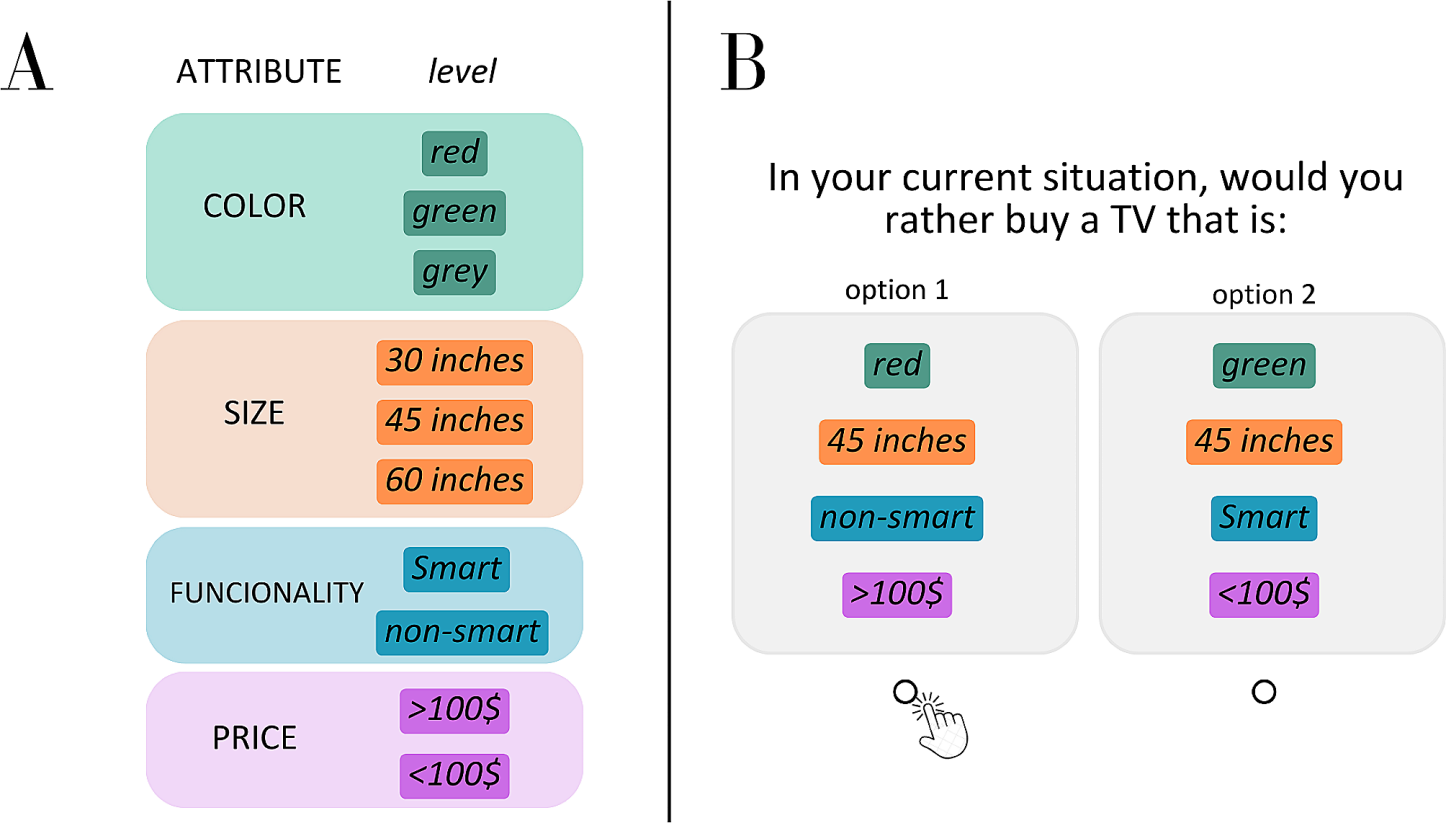
(**A**) Examples of different attributes which could interfere with a certain choice (e.g. color, size etc.). Each attribute is then defined by specific categories which caractherize them (they are named levels—e.g. for color—green or red; for size 30, 45 or 60 inches). (**B**) Example of paired choice combining different levels of different attribiutes in such experiment investigating the preference. Given a forced choice experiment, the respondent must have to choose one alternative even anyone is the perfect one for him/her. Then, they probably will choose one alternative which have the preferred levels of such attitbutes according to his/her opinion

Discrete Choice Experiments (DCEs) can be used to measure the value of each attribute (individual utilities) in choosing one alternative over others in clinical decision-making [13]. The preference for one given alternative over another depends on values built through knowledge, experience, and reflection [14]. A DCE is based on the random utility theory to measure the stated preferences of stakeholders. The preferences are measured by the valuation of attributes, which are characteristics that may influence the individual decision (e.g. colour). Each attribute is further defined by levels [15, 16], which are different manners of such characteristics appearing (e.g. green or red) (Fig. 1a). The attributes and their levels are combined in several possibilities to generate the profiles and tasks through the experimental design theory [17–19] (Fig. 1b). This study is pioneer using this methodology to understand dentists’ decisions and the actual weigh the scientific evidence represents on such regard.

Understanding the oral health professionals’ context and preferences captured through the attributes’ value may lead to informed planning of more relevant and effective implementation of evidence-based strategies in clinical practice by considering real-world data related to the consumption of evidence-based information. Consequently, we expect to improve the oral health professional’s compliance with clinical practice based on the current scientific evidence by maximizing strengthens and minimizing barriers related to that.

## Methods/Design

This protocol was written following the ISPOR Good Research Practices for Conjoint Analysis Task Force [15, 19], adjusted for a protocol (Additional file 1).

### Aim/Study Design

A cross-section DCE was designed to assess through the oral health professional’s perspective whether the available scientific evidence supporting a clinical alternative is considered in the decision-making process and how it is combined with other factors such as costs, patient needs, and/or dental associations or experts’ recommendations. Hypothetical situations will be created. Additionally, we intend to explore professionals’ preferences and choices associated with different interventions proposed in pediatric dentistry and supported by scientific evidence recently produced. Finally, we will check if labelling the options may influence professionals’ decisions despite the preferred attributes chosen in non-labelled alternatives. Due to these different aims, the experiment will be divided into different stages to contemplate each of the mentioned aims, and a specific DCE instrument will be designed for that.

### Research question and hypothesis

To satisfy the aims above, we defined two main research questions: (1) What is the relative importance of scientific evidence in oral health professionals’ decision-making? (2) What are the trade-offs between attributes involved in the decision to adhere to an evidence-based new clinical practice (intervention or diagnostic strategy which are not yet usual to professionals but recently fomented by scientific evidence).

### Participants

Our sample will comprise Brazilian oral health professionals who agree to participate in the study. As the application will be through an online platform, those who do not have access to a device such as a smartphone, tablet, computer and/or do not have access to the internet will not be able to participate.

The recruitment strategy will target dentists from both the public and private sectors. Health Departments from different states in Brazil will be contacted as facilitators to organize an application day for the public sector. Local events (congresses and commercial meetings) will be used to recruit participants from the private sector. Participants enrolled in this study will be invited to become citizen researchers and, after a training, apply the same instrument to their peers (other dentists) in their community. This approach will be used to achieve participants from different parts of Brazil and not only those who are close to research centers [20, 21].

Participants who consent to become citizen researchers will receive training to calibrate them both in ethical aspects and methodological aspects, guaranteeing the integrity and autonomy of the participants who will be recruited by them as well as the scientific confidence and rigor of the data collected [20, 22]. Individual and collective targets will be defined for the citizens research considering the number of dentists supposed to be achieved with this strategy of citizen science. A monitoring platform will be created [23] to motivate data collection and establish communication between investigators and citizen researchers.

The sample size will be calculated based on an arbitrary rule summing up 10 respondents for each attribute defined for the DCE and extra 50 participants [24]. By anticipating a maximum of six attributes in the experiment, we expect that a sample of 110 respondents would be needed to provide sufficient statistical power based on the rule described above. An additional number of participants will be increased in sample size for compensating possible a 20% non-respondent rate, totalizing an anticipated minimum sample of 132 dentists. After defining the attributes, this calculation will be adjusted, if necessary.

### Settings

The DCE instrument will be self-administered and delivered in a virtual environment. The computerized approach has already been used in previous studies measuring preferences [25]. This strategy is also reported as an alternative by the World Health Organization guidelines for conducting DCEs [26].

### Instrument properties

The template (electronic form) to be responded by the participants will include the Informed Consent Form, a preliminary questionnaire to capture participants’ information, and the DCE instrument by itself, containing the choice sets defined according to the steps described in the next sections.

The questionnaire will collect participants’ data such as gender, age, position (dentist, manager, coordinator), place of residence and work (Federative Unit of Brazil), year of graduation, and graduate courses (completed or not).

**Fig. 2:**
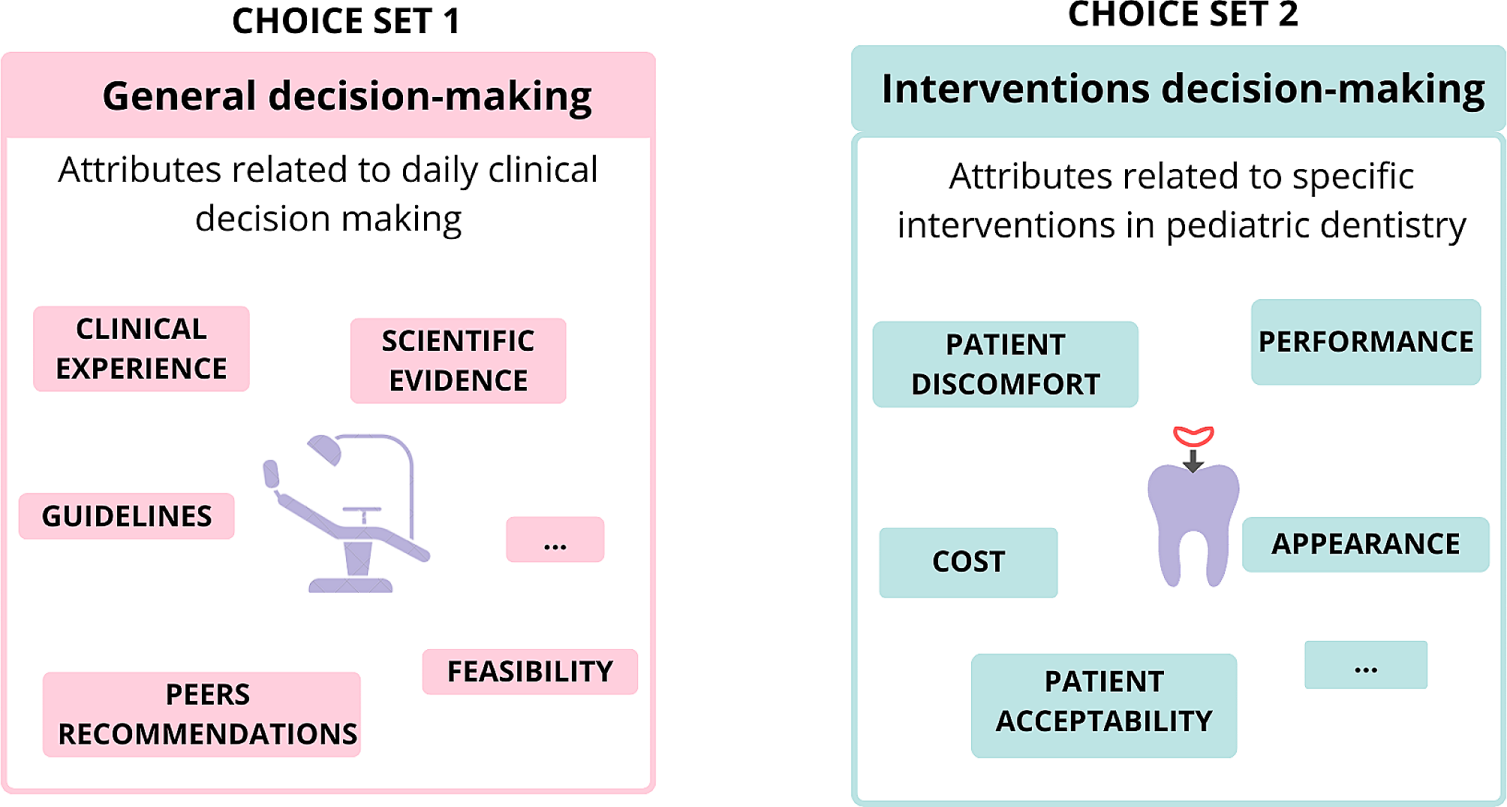
Two main choice sets and hypothetical factors that potentially affect the dentists’ decision

The DCE instrument will consist of two choices-sets with pairs of unlabeled alternatives (binary forced-choice experiment). Each choice-set comprises a different clinical decision context (Fig. 2).

The first one will focus on understanding the role of the attribute “scientific evidence” in the decision-making process. An experimental design will be created by combining different levels of attributes, resulting in hypothetical alternatives to measure that. The other set will focus on understanding specific attributes considered for choosing diagnostic and therapeutic interventions when such a decision is required in clinical practice. The interventions considered were related to caries diagnosis strategies and minimally invasive interventions. This set will be divided into three stages: (a) hypothetical alternatives combining levels of attributes, as the first set; (b) real alternatives, non-labelled, including the same attributes whose levels will be defined by pairs of interventions sourced by well-designed randomized controlled trials in the Brazilian context (Table 1), (c) the same alternatives from item “b”, but labelled. In this second set, the labelled and non-labelled alternatives (items “b” and “c”) will be displayed randomly to avoid generating an automatic association between them.

**Table 1:** Newly scientific evidence. Brief description of the randomized controlled trials that will be used to guide the DCE-interventions instrument, detailing the conditions and interventions tested in each of them

NCT or REBEC (PMID)*	Condition	Age (n)	Intervention group	Control Group	Follow-up (months)	Primary outcome	Secondary outcomes
02078453 (32,450,979)	Caries lesions detection and diagnosis^**(1)**^	3–6 (252)	Visual Inspection	Visual Inspection and bite-wing radiographs.	24	Surfaces with operative treatment needed	• New carious lesion; • Repairs needed; • Pain episodes; • Extractions; • Oral health-related quality of life • Economic
02473107 ( - )	Initial carious lesions detection ^**(2)**^	3–6 (260)	Detection and treatment based on all lesions severity	Detection and treatment based on advanced carious lesions	24	Surfaces with operative treatment needed	• Economic • Discomfort • Oral health-related quality of life
03520309 (32,615,237)	Carious lesions associated with restorations ^**(3)**^	3–10 (550 restorations)	Caries Around Restorations System (CARS) and International Caries Classification and Management System (ICCMS)	International Dental Federation (FDI)	24	Restorations with operative intervention needed.	• Restorations survival; • Repair needed; • Restoration substitution needed; • Carious lesion associated with restorations; • Pul inflamation symptons; • Economic; • Oral health-related quality of life.
02274142 (34,301,217)	Restorations survival^**(4)**^	3–10 (324 restorations)	Encapsulated GIC (Equia Fill®, GC Europe)	Hand-mixed GIC (Fuji IX®, GC Europe )	24	Restoration survival	• Economic
02789202 ( - )	Non-frankly cavitated carious lesions^(5)^	1–3 (100)	Silver Diamine Fluoride	Fluoride varnish	24	progression to dentin carious lesions	• Patient’s acceptability (VAS scale) • Economic
02569047 (33,176,756)	Occluso-proximal carious lesions^**(6)**^	5–10 (131)	Hall Technique	Atraumatic Restorative Technique	36	Restoration survival	• Discomfort • Patient’ acceptability • Occlusal Vertical Dimension • Oral health-related quality of life.
03005405 ( - )	Moderate carious lesion^**(7)**^	3–6 (101)	GIC sealant	GIC Restoration	24	Reintervention needed	• Discomfort • (Wong-Baker scale) • Economic
02377297 (32,758,684)	Restoring occlusal dentin caries lesions in primary molars [8]	4–8 (150)	Vitro Molar (nova DFL)/ Maxxion R (FGM)	Fuji IX Gold Label (GC Corp)	24	Restoration survival	• DMFT • Economic

NCT—Number ClinicalTrials.gov.

N—Sample Size.

GIC—Glass Ionomer Cement.

DMFT - Decayed, Missing due to caries, and Filled Teeth.

VAS—Visual Analogue Scale.

REBEC - The Brazilian Clinical Trials Registry / Registro Brasileiro de Ensaios Clínicos.

1. Pontes LRA, Novaes TF, Lara JS, Gimenez T, Moro BLP, Camargo LB, et al. Impact of visual inspection and radiographs for caries detection in children through a 2-year randomized clinical trial: The Caries Detection in Children-1 study. Journal of the American Dental Association. 2020;151 [6]:407 − 15 e1.

2. Martins IFN. Impacto da detecção de lesões iniciais e da avaliação da atividade de cárie em dentes decíduos: estudo controlado randomizado (CARDEC-02) com 1 ano de seguimento: Universidade de Sâo Paulo; 2017.

3. Moro BLP, Freitas RD, Pontes LRA, Passaro AL, Lenzi TL, Tedesco TK, et al. Influence of different clinical criteria on the decision to replace restorations in primary teeth. Journal of dentistry. 2020;101:103421.

4. Oliveira RC, Camargo LB, Novaes TF, Pontes LRA, Olegario IC, Gimenez T, et al. Survival rate of primary molar restorations is not influenced by hand mixed or encapsulated GIC: 24 months RCT. BMC Oral Health. 2021;21 [1]:371.

5. Viganó MEF. Is Silver Diamine Fluoride an option for treating non-frankly cavitated caries lesions on occlusal surfaces in toddlers?: findings on its efficacy and parents’ acceptance from a randomized controlled trial [dissertation]. 2021.

6. Araujo MP, Innes NP, Bonifacio CC, Hesse D, Olegario IC, Mendes FM, et al. Atraumatic restorative treatment compared to the Hall Technique for occluso-proximal carious lesions in primary molars; 36-month follow-up of a randomised control trial in a school setting. BMC oral health. 2020;20 [1]:318.

7. Rocha ES. O selamento é uma alternativa as restaurações para lesões de cárie moderada na superfície oclusal de dentes decíduos? : Faculdade de Odontologia; 2020.

8. Olegario IC, Ladewig NM, Hesse D, Bonifacio CC, Braga MM, Imparato JCP, et al. Is it worth using low-cost glass ionomer cements for occlusal ART restorations in primary molars? 2-year survival and cost analysis of a Randomized clinical trial. J Dent. 2020;101:103446.

**Fig. 3:**
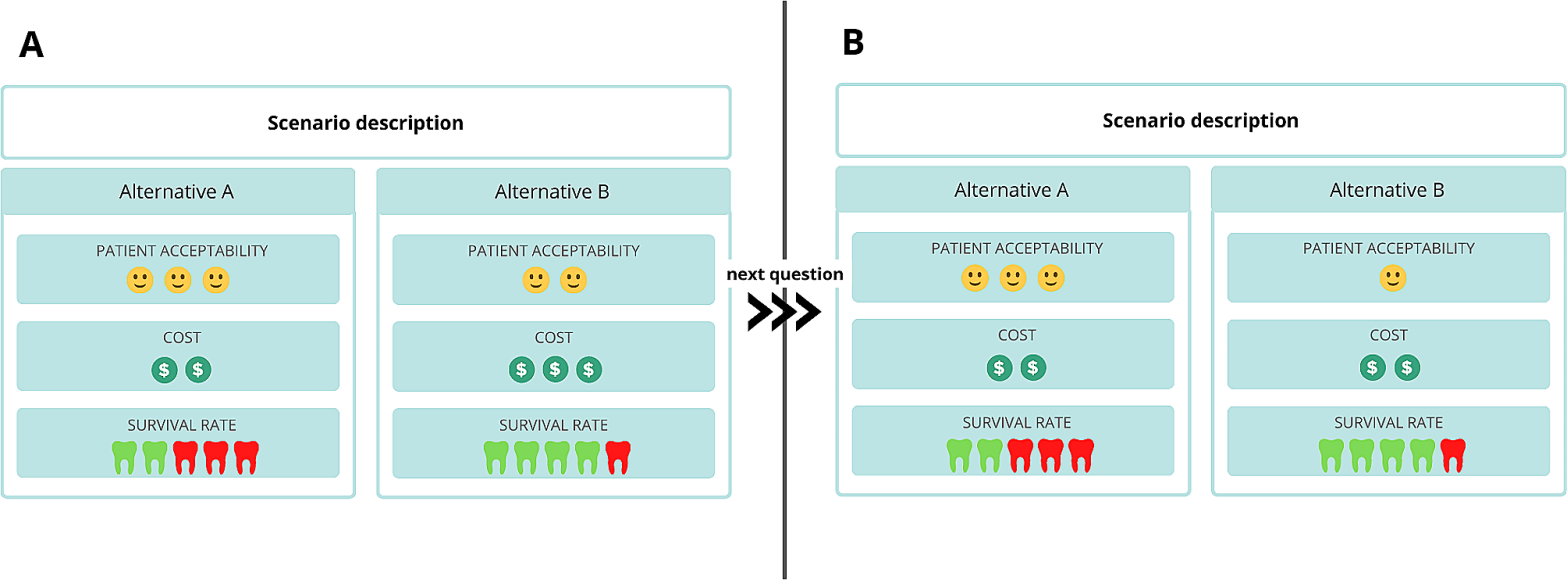
The flow of development of the Discrete Choice Experiment instrument

In the DCE instrument, participants will be provided a context (scenario) under the choice set followed by one of the binary unlabeled alternatives. Each alternative and the order for its appearance will be defined in the experimental design, as described in the next session (Fig. 3). Respondents must choose between the options available and click on “proceed”. Then, the context will not change until the final choice set is final, but the alternatives (levels) will vary. The choices made in the survey will be coded as chosen (1) or rejected (0). Data will be collected automatically in a specific database for the study.

### Instrument development

**Fig. 4:**
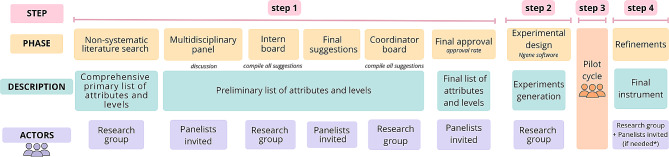
Presentation of sequential questions (A to B) with the scenario and hypothetical unlabeled alternatives. Note that when answering question A, question B is presented, varying hypothetical attributes “patient acceptability” and “cost”

The development of the DCE instrument was divided into four phases: 1-definition of attributes and levels; 2- determination of the experimental design (efficient combination of the attributes and attribute levels presented to the respondents), 3- pilot test of the preliminary instrument and 4-adjustments in the preliminary instrument to create the final version of the instrument to be used in the experiment (Fig. 4) [15, 19]. Each one of these phases will be detailed below.

Phase 1: **Definition of attributes and levels**—A non-systematic literature search will be performed, looking for all possible factors associated with the professional’s clinical decision-making (choice set 1) and related to specific interventions from Table 1 (choice set 2). Afterwards, a discussion in the research group will be guided to generate a comprehensive primary list of all potential attributes and their variation in potential levels for each choice set. Then, a multidisciplinary panel will be formed, including stakeholders from different Brazilian regions, aiming to guarantee national representativeness for discussing and finally defining the definitive list of attributes and levels. Ideally, the panel will comprise the following representatives: dentists who work in private clinical practice, dentists who work in public practice, undergraduate students, health service managers, researchers who work with knowledge translation, researchers who work with evidence-based practice, and opinion leaders in Dentistry. The preliminary list with all attributes and their levels, definitions, justification, and references for each attribute will be presented, and a discussion will be opened regarding the range of levels, understanding of the attribute, and whether other attributes should be added. Besides, suggestions to reformulate levels and attributes should be accepted.

The multidisciplinary panel will be recorded, and a qualitative thematic analysis [27, 28] will be performed to explore further possible differences in the discourses of selected stakeholders and the respondents’ views. The discussion will be verbatim transcribed using the voice typing tool from Google Docs [29], and the authors of the speeches will be anonymized for the publication and dissemination of the results. An independent investigator will be invited to carefully read the transcripts while hearing/watching the recording to ensure that the transcription was completed in full. Qualitative data analysis will be described further.

By the end of the panellist discussions, the coordinator board (MMB, FMM, DPR, GMM, ACFL) will be gathered to refine the list generated, preserving the previously given contributions. The final attributes and levels list will be finally sent to all panellists, and each level’s approval rate will be registered. To be approved, final levels and attributes must be approved by the most representatives in the panel (at least 80% of approval). If this approval rate is not achieved, all panellists’ views about such a specific disapproved level will be reconsidered.

Phase 2**—Determination of the experimental design**—At this stage, the aim was to produce a number of pairs of alternatives, combining the levels of the attributes. The experimental design is a manner to combine these pairs for each choice set in the most efficient arrangement of the pairs of alternatives, aiming not to use all possible combinations (cognitively impossible), but a number enough to permit the preference to be measured. The more attributes and levels (more sets of choices) used, the greater the complexity of the experiment and, consequently, the more significant unobserved variability we must consider in the analysis [18]. A list with minimum but enough combinations will be generated at the end. We will exclude alternatives with implausible combinations of unbalanced choice levels and sets to adjust the experiment, ensure the plausibility of the choices, and reduce hypothetical bias (when the hypothetical nature of the questions results in biased answers). The design efficiency (Efficiency D) will be calculated to determine if the number of pairs can assess the possible effects (preference) intended to be measured. These calculations will be performed using the Ngene software [17, 19, 30].

Phase 3—**Pilot test of the preliminary instrument** - A pilot study will be conducted to determine whether the DCE instrument is appropriate for the main investigation. As described above, an electronic form will be created for the final version. However, specific questions about the difficulty level and the time needed to answer the questions in each block will be inserted after each choice set. For this purpose, no pre-set sample size is required [17]. A minimum convenience sample of 10 dentist volunteers is foreseen, but depending on the observations, more respondents can be included in this phase. Besides completing the form, the pilot respondents will be invited to share their difficulties and opinions with the coordinator board to create suggestions for improvement for the experiment. In case the pilot instrument is acceptable and does not need adjustments, this sample will be considered automatically as part of the main sample - an internal pilot study [31, 32]. In case of amendments are needed, and a change in the instrument or the experimental design is mandatory, an external pilot will be considered [32].

Phase 4—**Adjustments in the preliminary instrument –** Based on inputs of the pilot respondents regarding cognitive exhaustion, reasonability and comprehensibility of the tests, any amendment may be done in the attributes and levels, number of alternatives, experiment format or presentation. Depending on the type of the request/query, one or more phases described above will have to be redone, and eventually, a new pilot study may be necessary to retest the changes and produce a final experiment to be tested in the whole main sample.

### Data Collection—Experiment

Recruited participants will have access to the final DCE instrument invited by the research team or any citizen researcher. After reading and agreeing to participate, they will answer both choice settings, following the structure of each one previously described in this paper.

All collected data will be stored in a cloud (Google Drive) and anonymized by the study coordinator in the team (GMM) using random numbers. Only this researcher will access the identified data and the identified informed consent forms. The research team will work on the anonymized dataset. At the end of the study, all collected anonymized data will be downloaded for data analysis, and any record from any virtual platform will be deleted. Finally, data will be available online in an appropriate institutional repository after the publication of the final results. Missing data will be identified, and the method of conditional imputation will be used, considering an appropriate regression model according to the type of variable to be imputed.

### Analysis plan

An analysis plan for this protocol is prepared and made available in the DMPHub, using the DMPtool [33].

#### Qualitative analysis

The framework method will be used for performing a qualitative content analysis [34]. Data organization and analysis will follow a predefined sequence [28]: (1) editing material, which comprises the organization of the data collected and the creation of the subgroups (such as private clinical practice dentists, and knowledge translate researchers); (2) free-floating reading, reading the collected data freely, with no intention of categorizing to understand the general context; (3) construction of the units of analysis, reading each excerpt and conducting the first preliminary codes of meaning and group together the speeches that suggest the same meaning (Maxqda® software can assist in the coding); (4) identification of cores of meaning, re-reading of the previously identified grouped speeches, to give them a code (entitling them), (5) consolidation of categories, refining the codes; (6) discussion of the topics with the group and the literature; (7) validity, considering the research question.

#### Quantitative and statistical analysis

The characteristics of the respondent sample will be examined against the known characteristics of the population whose preferences researchers may want to generalize. We will use appropriate tests to examine the hypothesis that the respondent sample has been drawn from the desired population. Additionally, we will compare the sample recruited by the traditional and citizen science approach to verify any possible differences and, eventually, explore or adjust any subsequent analysis.

The validity of the data will be checked considering response error (included in the set of choices to detect internal validity failures). For that, we will analyze the occurrence and the frequency of the respondents who always or nearly always choose the alternative with the best level of one attribute, and preferences dominated by a single attribute. Any failure of internal validity detected will be statistically controlled in further analyses.

From the main DCE, we expect to estimate the strength of preferences for the attributes included in the survey. We also expect to demonstrate how choice probabilities may vary with changes in attributes or attribute levels.

At a first attempt, the probability of choice for each attribute (and its respective levels) may be calculated. Data generated by the DCE will be coded for further analysis. We will adopt the dummy-variable coding as the option for categorical coding of attribute levels [35]. Conditional logistic regression and latent-class finite-mixture models will be used to estimate average preferences (the probability of choice) across a sample considering two alternatives and attributes and levels, as well as heterogeneous effects on choices across a finite number of groups or classes of respondents. We will assume that multiple observations from the same respondent are independent [36]. Another purpose of such statistical analyses might be to estimate how preferences vary by individual respondent characteristics. Multinomial regression models will be used to relate respondents’ choices to respondents’ personal and professional characteristics (respondents’ profiles) [36]. For goodness-of-fit, the likelihood ratio chi-square test will be calculated for each model to provide a way to determine whether the inclusion of attribute-level variables significantly improves the fit of the model compared to a null model.

Subgroup analysis considering different federative units and types of practice (public or private) will be performed to explore possible differences among different groups that demand future individualization of implementation processes.

## Discussion

Rigorously produced science provides reliable information to guide clinical decisions [10, 37]. However, there is frequently a gap between scientific evidence produced and healthcare provided [12, 38], taking time [2] for the implementation of research findings into clinical practice (when it occurs) [12, 39, 40]. Besides the unpreparedness of health professionals to critically assess scientific literature for implementation in practice and to deal with it independently [34], their resistance to change, allegiance to precursor thoughts, personality traits, and beliefs [12] may contribute to this gap. It is possible that scientific evidence, which we thought was an established crucial criterion, may not be as relevant to dentists as other factors, such as treatment complexity, patient expectations, etc. Therefore, guiding evidence-based practice on the academic belief that such practice is relevant may not be the more efficient way to implement the evidence into clinical practice.

Using the methodology expected for a DCE, it is possible to capture the importance of different attributes in a group of respondents without objectively asking them which attribute they prefer. When a respondent is exposed to the experiment, the hypothetical alternatives lead him/her to choose the alternative more compatible with his/her expectations. However, if there is no ideal (perfect) alternative to him/her, he/she will mostly opt for the alternative that presents the ideal level of the most valued attribute for him/her. Considering the example given in Fig. 1, if the respondents consider cost as a very relevant aspect, they will tend to always opt for the alternative in which “low cost” (<$100) appears. They will have to opt for a second preferred option only when this option does not appear. Therefore, they do not have to say: “I prefer this or that”, but they intuitively will point out in one common direction. The repetition of this pattern will finally reflect their preference. Such a strategy has often been used to measure preferences for attributes of medical interventions, characterizing the preference by attribute importance [34].

By collecting the information using this more intuitive strategy, we believe such an assessment may reduce the choice-supportive bias on our findings. Several studies have observed that choice-supportive bias has the potential to affect future choices [41]. Previous memories, for example, related to previous knowledge of the “expected’ or “correct” answer, could influence the dentist to opt for such an expected answer (e.g., evidence-based concepts). When all attributes, including this one, are displayed together, the attribute is not in focus. Since it will be combined more naturally with the others than a unique binary question, the respondents may be more prone to not “force” non-real answers.

This study will investigate attributes that guide the choice of dental professionals in Brazil. Thus, if “non-important” attributes are valued at the expense of those who can bring decisions with greater security and benefits, future interventions can be planned to directly impact them and guide the implementation of recently produced evidence more efficiently. Bring a parallel to marketing strategies; understand the dentist as a consumer. If the marketing survey shows consumers prefer a box, no marketing strategy will consider selling a product in a bag. Coming back to evidence-based practice in health care, we should use the “appeal” that consumers or professionals are prone to adopt and customize the implementation of such evidence in clinical practice or policies.

This assessment also aims to identify possible professionals’ choice trends based on the name of the intervention rather than its characteristics. Disruptive scientific evidence may require a paradigm shift in clinical practice. Different types of cognitive bias may influence decisions and difficult changes in this process [41, 42]. Certainly, difficulties in the implementation process may result from that. Due to the reasons discussed above, the DCE may also represent an opportunity to show if the preferred (or most valued) attributes reflect the actual choice of known/traditional interventions compared to the disruptive evidence-based alternatives. Recent studies have shown that dental radiographs for detecting caries do not bring additional benefits and may cause overdiagnosis, false-positive results and lead-in time bias [43, 44]. However, it is current practice and advocated in some Pediatric Dentistry guidelines [45–47]. Depending on dentists’ allegiance or choice-supportive memories, they may resist change. This is a real example of recognizing what dentists consider in their decision-making process to make a change feasible.

Usually, DCEs are conducted with a sample of respondents representing the “possible consumers” we intend to assess the preferences. Since the idea is to guide the implementation of recently produced science into clinical practice in Brazil, the focus groups of our experiments are supposed to be Brazilian dentists. However, important differences have been observed among different regions in Brazil [48]. Such a deal will probably be minimized since, as a multicenter study, representatives from different Brazilian regions comprise our research group. Additionally, we opted for a citizen science strategy [20], which will certainly disseminate the researchers’ action in each area and may result in a more accurate representation of different respondents’ profiles and possibly reflect different pattern preferences. Even preferences may vary from country to country; this pioneering study may produce primary information on how the scientific evidence has been adopted among health professionals and inspire adaptations to other contexts.

From a wider perspective, such methodological options may lead to findings that may feed an implementation process in a more representative, inclusive, and equitable way and, in the future, can still contribute to better outcomes for the population’s health in general.

### Electronic supplementary material

Below is the link to the electronic supplementary material.

Supplementary Material 1

## Data Availability

All data, including the questionnaires, all resources, and intermediate parts, will be available at https://osf.io/bhncv. The final data will be available as open data via the University of Sao Paulo online data repository.

## Abbreviations

DCE: Discrete Choice Experiment
