# Supplementary material for: How different attributes are weighted in professionals’ decision-making in Pediatric Dentistry—a protocol for guiding discrete choice experiment focused on shortening the evidence-based practice implementation for dental care
Source: BMC Oral Health. 2024 Apr 19;24:474. doi: 10.1186/s12903-024-04090-3 (PMC11031987; doi:10.1186/s12903-024-04090-3)
Supplement: Supplementary file 1 — Supplementary Material 1 [file 12903_2024_4090_MOESM1_ESM.pdf]

ISPOR
Improving healthcare decisions

Conjoint Analysis Applications in Health—a Checklist

Section	Page
1) Research Question	8
2) Attributes And Levels	11
3) Construction Of Tasks	12
4) Experimental Design	13
5) Preference Elicitation	13
6) Instrument Design	11-14
7) Data-Collection Plan	15
8) Statistical Analyses	15,16
9) Results And Conclusions	protocol
10) Study Presentation.	protocol

From Bridges, J. F., et al. (2011). "Conjoint analysis applications in health--a checklist: a report of the ISPOR Good Research Practices for Conjoint Analysis Task Force." Value Health 14(4): 403-413.
